# Automated cell type discovery and classification through knowledge transfer

**DOI:** 10.1093/bioinformatics/btx054

**Published:** 2017-01-31

**Authors:** Hao-Chih Lee, Roman Kosoy, Christine E Becker, Joel T Dudley, Brian A Kidd

**Affiliations:** 1 Department of Genetics and Genomic Sciences, Icahn School of Medicine at Mt. Sinai, New York, NY, USA; 2 Icahn School of Medicine at Mt. Sinai, Institute for Next Generation Healthcare, New York, NY, USA

## Abstract

**Motivation:**

Recent advances in mass cytometry allow simultaneous measurements of up to 50 markers at single-cell resolution. However, the high dimensionality of mass cytometry data introduces computational challenges for automated data analysis and hinders translation of new biological understanding into clinical applications. Previous studies have applied machine learning to facilitate processing of mass cytometry data. However, manual inspection is still inevitable and becoming the barrier to reliable large-scale analysis.

**Results:**

We present a new algorithm called **A**utomated **C**ell-type **D**iscovery and **C**lassification (ACDC) that fully automates the classification of canonical cell populations and highlights novel cell types in mass cytometry data. Evaluations on real-world data show ACDC provides accurate and reliable estimations compared to manual gating results. Additionally, ACDC automatically classifies previously ambiguous cell types to facilitate discovery. Our findings suggest that ACDC substantially improves both reliability and interpretability of results obtained from high-dimensional mass cytometry profiling data.

**Availability and Implementation:**

A Python package (Python 3) and analysis scripts for reproducing the results are availability on https://bitbucket.org/dudleylab/acdc.

**Supplementary information:**

Supplementary data are available at *Bioinformatics* online.

## 1 Introduction

High-throughput, high-dimensional cytometry is one of the most valuable tools for basic and clinical immunology. Advances in this technology over the last decade now provide simultaneous measurements of dozens of proteins at single-cell resolution (Bandura *et al.*, 2009; Spitzer and Nolan, 2016). Mass cytometry by time-of-flight (CyTOF) provides a powerful new tool for studying cellular diversity and dynamics by measuring up to 50 markers per cell. Many recent studies highlight the utility of CyTOF for enabling novel discovery and understanding in multiple domains of immunology, including mapping cell subset heterogeneity and specificity in response to various pathogens (Newell *et al.*, 2012, 2013), precise elucidation of cellular networks and biochemical pathway activation following drug perturbation (Bendall *et al.*, 2011; Bodenmiller *et al.*, 2012), as well as new understanding of cellular trafficking and tissue localization (Wong *et al.*, 2016a, b). However, the high number of measures and complexity of the resulting data restrict manual exploration and present challenges for both the analysis and biological interpretation of CyTOF data (Newell and Cheng, 2016). New tools that automate the data analysis are needed to realize the full potential of CyTOF for biological discovery and translational applications.

A number of studies have focused on applying or developing algorithms to address the data analysis and interpretation challenges arising from CyTOF data. One early approach applied machine learning techniques to detect clusters of similar immune cell types in high dimensional space (Aghaeepour *et al.*, 2013; Qiu *et al.*, 2011). More recently, researchers have used network analysis techniques to assist the identification of known and novel cell populations (Levine *et al.*, 2015; Samusik *et al.*, 2016; Shekhar *et al.*, 2014). In concert with these analytical advances, a number of studies have developed software tools to organize and visualize the high-dimensional cytometry data (Amir *et al.*, 2013; Shekhar *et al.*, 2014; Van der Maaten and Hinton, 2008). Yet, to date, the available computational tools still require substantial manual manipulation to extract biological findings and interpret the data. These manual steps create a major limitation for exploring the full dataset and taking advantage of the large number of markers in CyTOF.

One of the biggest challenges for interpreting mass cytometry data is how best to annotate individual cells with canonical cell types. This difficulty arises from (i) uncertainty in defining cell types based on more than a handful of markers and (ii) the absence of biological information as an input for machine learning techniques. Current approaches require substantial manual inspection that impedes the analysis workflow, underutilizes the full value of the high-dimensional data, and ultimately reduces the scientific insights that can be gained from each study. Here we address the cell annotation challenge through a novel computational method that greatly facilitates the organization and interpretation of mass cytometry data through automated transfer of biological knowledge.

Our method automates cell annotation by using biological knowledge as an input parameter to a novel machine learning approach: **A**utomated **C**ell-type **D**iscovery and **C**lassification (ACDC). ACDC provides enhanced visualization and automated classification of canonical cell populations, as well as augments the discovery of novel populations from mass cytometry data. ACDC represents a new framework that seamlessly integrates all the pieces to automate the process for estimating occurrences of canonical cell populations. We evaluated ACDC using three benchmark datasets (AML (Levine *et al.*, 2015), BMMC (Bendall *et al.*, 2011; Levine *et al.*, 2015) and PANORAMA (Samusik *et al.*, 2016), for which manual gating information was available to provide a ‘ground truth’ reference.

## 2 Methods

Annotating individual cells requires reconciling the vast amounts of single cell information collected through high-throughput cytometry with our prior knowledge. To illustrate this point, it is well established that a CD4+ T-cell is identified based on high levels of CD3 and CD4 and simultaneously having low expression level of CD8. We designed ACDC to take advantage of the biological knowledge that humans have accumulated and integrate this information with machine learning algorithms to automate the annotation of mass cytometry data.

**Fig. 1:**
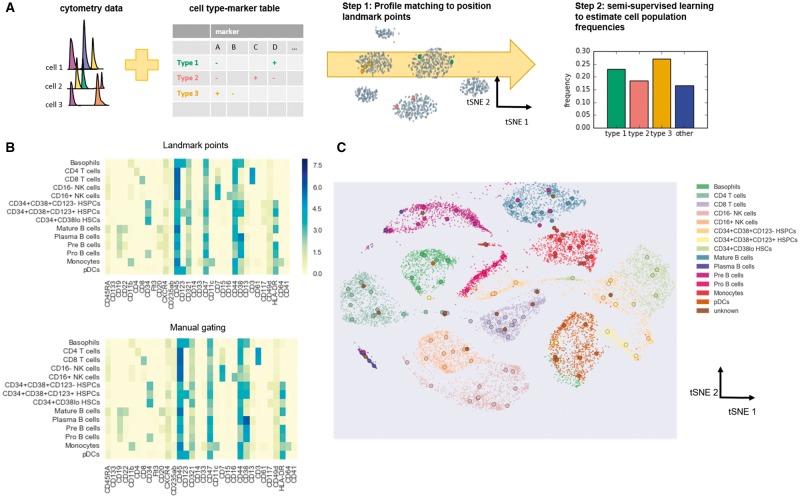
ACDC algorithm design and validation. (**A**) Schematic diagram showing the work flow of ACDC. (**B**) Heat maps showing the average marker intensity of landmark points and manually gated populations from the AML dataset. (**C**) tSNE visualization of landmark points (large circles) and manually gated populations (dots)

To combine our prior biological frameworks with new data, the ACDC approach involves two steps (Fig. 1A and Supplementary Fig. S1**).** First, ACDC converts a user-specified table of markers and cell labels into landmark points that represent fingerprints for specific cell types in the high-dimensional space. Second, ACDC implements semi-supervised classification via random walks (Grady, 2006) to collect information from all the landmark points and classify events at the single-cell resolution. With ACDC, prior knowledge of canonical cell types is explicitly encoded in the user-specified table, transformed into landmark points and eventually fed into a semi-supervised learning algorithm. We summarize the workflow of ACDC in the following:

1. *Inputs*: measured mass cytometry events and a user-specified table of markers to cell types.
2. **Generate** landmark points by score matching and un-supervised clustering. (Section 2.1)
3. **Classify** single-cell events by semi-supervised learning. (Section 2.2)

Study design and evaluations are presented in Section 2.3.

### 2.1 Generate landmark points

#### 2.1.1 Design of cell type-marker table

A cell type-marker table is a data matrix $s(c_{j},\;m_{k})$ whose value is either 1 (present, +), -1 (absent, -) or 0 (do not consider), where $c_{j}$ is the *j*th cell type and $m_{k}$ is the *k*th marker (Supplementary Table S1–S3). The cell type-marker table allows users to customize cell types to be detected by linking these canonical cell types to their marker profiles. For example, CD4+ T-cells are known to have high expression level of the surface markers CD3 and CD4 and low expression level of CD8. Therefore, CD4+ T-cells are described as CD3+/CD4+/CD8- cells. As another example, B-cells can be referred to as CD19+/CD3-cells. ACDC converts the user specified cell type-marker table into landmark points in the high-dimensional space.

#### 2.1.2 Design of the score function

We designed the score function to match a mass cytometry event with a single cell type. Intuitively, the chance a measured event belongs to a canonical cell type is determined by the extent that the intensity profile of a cluster matches one of the pre-specified profiles. We formulated the degree of matching as the posterior probability that a marker is in the activated/inactivated state. To be precise, we first fit a two-mode Gaussian mixture model $P_{k}\;$to the *k*th marker’s intensity distribution. While the marker intensity is one dimensional, we identified the mode of high/low intensity as the activated/inactivated state of this marker. The score of assigning an event $w_{i}\;$to a cell type $c_{j}$ is then defined by

$$f(w_{i},\;c_{j})=\;{min}_{k\;if\;s\left(c_{j},\;m_{k}\right)\neq 0}P_{k}\left(s\left(c_{j},\;m_{k}\right)||w_{ik}\right)$$

where $P_{k}\left(s\left(c_{j},\;m_{k}\right)||w_{i}\right)$ is the posterior probability of the *k*th marker is in state $s\left(c_{j},\;m_{k}\right)\;$and $w_{ik}$ is the intensity of the *k*th marker in an event $w_{i}$. The minimum is taken over all specified markers to ensure that all requirements are satisfied. In practice, cell types specified by a user might not be exhaustive. To detect those unspecified cells, we added an ‘unknown’ type whose score is defined by

$$f\left(w_{i},unknown\right)=\;1-\underset{c_{j}}{\max}{(min}_{k\;if\;s\left(c_{j},\;m_{k}\right)\neq 0}P_{k}\left(s\left(c_{j},\;m_{k}\right)||w_{ik}\right)).$$

This quantity represents the level of uncertainty in our current knowledge since its high value indicates the low probability of assigning any specified cell types to the event $w_{i}$.

Though $P_{k}$ can be directly evaluated by the Gaussian mixture model, such posterior probability might not be monotonic if the Gaussian mixture model has modes of unequal variances. We instead used an approximated function

$${\tilde{P}}_{k}\left(s=1||w\right)=\frac{\;\;\text{exp}\;(\left(w-a\right)\times b)}{1+\;\;\text{exp}\;(\left(w-a\right)\times b)}$$

where a is the critical point that $P_{k}\left(s=1||w_{i}\right)=\;P_{k}\left(s=0||w_{i}\right)$ and b is the slope of the posterior probability at this critical point. Both a and b can be computed from the means and variances of the two-mode Gaussian mixture model.

#### 2.1.3 Unsupervised clustering

Community detection (Girvan and Newman, 2002) was used due to its superior performance in clustering mass cytometry data (Levine *et al.*, 2015). The community detection aims to find a set of assignments $c_{i}\;$that maximize the modularity *Q* defined by

$$Q=\frac{1}{2m}\sum_{ij}\left[W_{ij}-\frac{s_{i}s_{j}}{2m}\right]\delta (c_{i},c_{j})$$

where $W_{ij}$ is the weights between *i*th node and *j*th node, $s_{j}=\sum_{k}W_{kj}$ and $m=\sum_{ij}W_{ij}/2$. $\delta \left(u,v\right)$ is the Krnoecker delta function that takes values of 1 when u= v and 0 otherwise. $c_{i}$ is the community assignment of *i*th node. We used the recommended setting to generate the weight matrix $W_{ij}$ based on 30-nearest neighbor graph and Jaccard similarity (Levine *et al.*, 2015).

#### 2.1.4 Landmark point generation

To generate landmark points, we partitioned the whole dataset into subsets $S_{j}=\{w_{i}\;|\;f\left(w_{i},\;c_{j}\right)>1/2\}$. Landmark points were defined as the centers of clusters identified by community detection in each subset.

### 2.2 Single-cell classification by semi-supervised learning

#### 2.2.1 Classification by random walkers

We implemented semi-supervised classification via random walks (Grady, 2006) for classifying events at the single-cell resolution. Briefly, semi-supervised classification via random walks evaluates the probability that a data point x belongs to class c as the chance of a random walker, starting from the data point x, first reaches a landmark point l of class c when navigating the network. Theoretical derivation shows this probability satisfies the Laplace equation, i.e.

∇P(x|c)=0,

with the boundary conditions P(l|c)=1 if a landmark point l of class c and P(l|c)=0 if a landmark point l of other classes. The numeric value of $P\left(x|c\right)$ at every data point can be solved as a boundary value problem. In our implementation, we used 10-nearest neighbors to construct such a data network.

#### 2.2.2 Processing experiments with multiple replicates

A common experimental design with mass cytometry data is to measure multiple biological examples of a particular type (e.g. organism, tissue, treatment condition) in one experiment. To classify data from these replicate samples on a common basis, we computed a common set of landmark points using pooled data of all replications and then classify each replication independently with the same landmark points. Cell frequencies were then estimated by counting the classification results.

### 2.3 Study design and benchmarking

#### 2.3.1 Validation datasets

We used three public benchmark datasets. BMMC dataset is a mass cytometry dataset collected from healthy human bone marrow (Bendall *et al.*, 2011). While 34 parameters were originally measured, the publically available dataset reduced to only 13 markers, and the resulting dataset included 24 populations gated based on these markers (Levine *et al.*, 2015). AML dataset is also collected from healthy human bone marrow (Levine *et al.*, 2015), and consists of 32 markers and 14 manually gated classes. PANORAMA dataset is a recently published dataset that provides replicative measurements of mass cytometry data from mice, where 24 cellular populations were gated based on 38 surface markers (Samusik *et al.*, 2016). Three experts independently gated the cellular populations in the PANORAMA dataset and only the consensus part of the gating was retained. All event measurements were transformed by $\sinh^{-1}\left((x-1)/5\right)\;$before further processing (Samusik *et al.*, 2016).

Cell type-marker tables were generated according to previous studies (Bendall *et al.*, 2011; Levine *et al.*, 2015; Samusik *et al.*, 2016). The cell type–marker tables of the BMMC and AML dataset were generated based on their gating hierarchy provided on Cytobank (Supplementary Table S1 and S2). In BMMC dataset, erythroblast, megakaryocyte platelet and myelocyte were merged as an unknown population since negative markers exclusively define these cells. For the PANORAMA dataset, the cell type-marker table was generated based on the divisive marker tree with minor changes (Samusik *et al.*, 2016) (Supplementary Table S3). We excluded HSC cells and pro B cells as unknown types since their defining markers cannot be determined from the reported divisive marker tree.

#### 2.3.2 Baseline methods

We implemented (i) score-based classification; and (ii) phenograph clustering (Levine *et al.*, 2015) for performance benchmarking. The score-based classification assigns event $w_{i}$ to the class $c^{*}$ that maximizes the score, i.e.

$$c^{*}=argmax_{c}f\left(w_{i},c\right),$$

where f is the designed score function. For the phenograph clustering, data was first clustered by community detection and then all events within a cluster were assigned to a manually gated cell type of highest frequency in this cluster. This method was implemented as a counterpart of estimating population frequencies by unsupervised clustering.

#### 2.3.3 Evaluation metrics

We applied three metrics to evaluate the performance on estimating cellular population frequencies. Given two normalized histograms $h_{1}$ and $h_{2}$, generated by counting the number of each cellular category classified either manually or automatically, the maximum error is computed by taking maximum of absolute errors on all components. To be precise, the maximum error is defined by

$$d\left(h_{1},h_{2}\right)=\underset{i}{\max}{|h}_{1,i}-h_{2,i}|,$$

where $h_{1,i}$ and $h_{2,i}$ are *i*th elements of histograms $h_{1}$ and $h_{2}$, respectively. The Canberra distance is defined by

$$d\left(h_{1},h_{2}\right)=\sum_{i}\left|h_{1,i}-h_{2,i}\right|/\left(h_{1,i}+h_{2,i}\right).$$

This distance is chosen to estimate the capability of capturing rare populations since it gives higher penalty on the low-frequency populations. Lastly, the intersection distance, defined by

$$d\left(h_{1},h_{2}\right)=1-\underset{i}{\mathrm{sum}}\min\left(h_{1,i},h_{2,i}\right),$$

measures the difference between the common area underlying two histograms and 1, which is the largest possible common area. The intersection distance reflects the accumulative errors in all populations.

The accuracy of classifying single-cell events is measured by the F1-score, which reflects the harmonic mean of precision (purity) and recall (yield),

$$F_{i}=2\times \frac{P_{ii}\times R_{ii}}{P_{ii}+\;R_{ii}},$$

$$P_{ij}=\frac{C_{ij}}{\sum_{k}C_{ik}},\;R_{ij}=\frac{C_{ij}}{\sum_{k}C_{kj}},$$

where $C_{ij}$ is the number of events classified as population *i* that belongs to the manually gated population *j*.

#### 2.3.4 Confidence estimation

For validation on AML and BMMC datasets, the confidence level was estimated using 5-fold cross validation while keeping the percentage of samples for each class unchanged. For the PANORAMA dataset, confidence level was estimated as the standard deviation over samples.

#### 2.3.5 Measuring tightness of clusters

We used the silhouette coefficient to measure the tightness of a given cluster (Rousseeuw, 1987). The silhouette coefficient measures how similar a datum is to its own cluster compared to the other clusters. For the *i*th datum, silhouette coefficient of this datum is defined as

$$s_{i}=\frac{{b_{i}-a}_{i}}{max(a_{i},\;b_{i})},$$

where $a_{i}$ is the average Euclidean distance from this datum to other members of the same cluster, and $b_{i}$ is the lowest average distance from this datum to members of other clusters. The silhouette coefficient ranges from -1 to 1 while a negative silhouette coefficient indicates a datum is closer to other clusters than its own cluster.

## 3 Results

### 3.1 ACDC helps visualization of mass cytometry data

To test whether the detected landmark points represent the corresponding cellular populations, we first applied ACDC to the AML and BMMC datasets. In the AML dataset, ACDC identified every population highlighted in the study and showed virtually no difference with manual gating (Fig. 1B). The one exception was a population of CD34 + CD38 + CD123+ HSPCs that showed a lower average intensity of CD123 in ACDC than with manually gating. To examine how landmark points depicted cellular populations, we used tSNE (Van der Maaten and Hinton, 2008) to map cellular measurements sampled from the manually gated populations onto a two-dimensional space and displayed the detected landmark points in their respective coordinates (Fig. 1C). The tSNE projection also supports the observation that landmark points detected by ACDC fall within their corresponding cluster of cells. We found similar results in the BMMC dataset (Supplementary Fig. S2). These results confirm that landmark points can locate cellular populations as accurate as manual gating.

### 3.2 ACDC classifies canonical cell populations as accurate as human experts

**Fig. 2:**
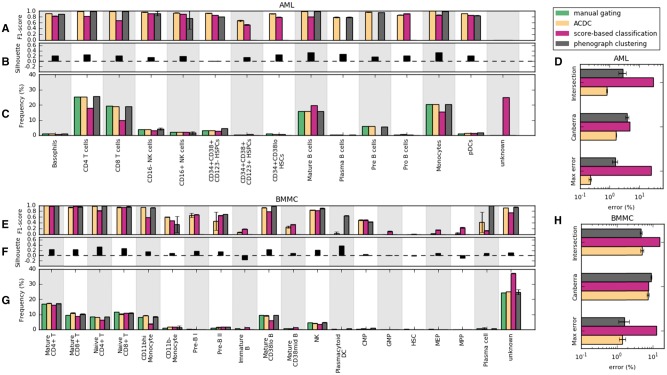
Validation on AML and BMMC datasets. (**A, E**) Classification accuracy of ACDC (yellow bars), score-based classification (purple bars), and phenograph clustering (gray bars) evaluated by F1-score. (**B, F**) Silhouette coefficients of manually gated populations show cluster tightness. (**C, G**) Comparison of population frequencies estimated by the 3 methods versus manual gating (green bars). (**D, H**) Errors in estimating population frequencies. Error bars reflect the standard deviations of the accuracy estimates from the cross-validation trials described in Section 2.3.4

Although landmark points aid the exploratory analysis of mass cytometry data, the focus of this study was to evaluate whether landmark points classify events accurately at single-cell resolution. For comparison, we implemented two alternative classification methods: (i) a score-based classification that assigns an event to the class of the highest score and (ii) phenograph (Levine *et al.*, 2015) clustering combined with manual gating to annotate each cluster. Overall, ACDC achieved comparable accuracy (92.9 ± 0.5% for BMMC and 98.3 ± 0.04% for AML) on classifying single-cell events with phenograph clustering (93.6 ± 0.7% for BMMC and 96.5 ± 0.7% for AML) and significantly improved the score-based classification method (78.1 ± 0.03% for BMMC and 68.4 ± 0.1% for AML). We also analyzed the classification performance for each cell type (Fig. 2A and E). In the AML dataset, ACDC achieved a median F1-score of 0.93, compared with 0.84 for the score-based classification and 0.83 for the phenograph clustering. We observed a lower performance of ACDC in the BMMC dataset (median F1-score of 0.60, compared with 0.63 for the score-based classification, and 0.55 for the phenograph clustering) due to the difficulty in detecting rare populations with frequencies less than 0.5%, such as GMP, HSC, MEP and MPP. However, low silhouette coefficients suggest that these rare populations may not form well-defined clusters (Fig. 2B and F and Supplementary Fig. S3). Both the score-based and phenograph clustering methods also failed to identify these rare populations due to a lack of representative data for these cell types.

### 3.3 ACDC estimates frequencies of canonical cell populations as accurate as human experts

We next addressed the practical issue of estimating the frequency of a cell population. When applied to the AML and BMMC datasets, ACDC and the phenograph clustering gave estimates comparable to the manually gated ones while the score-based classification method overestimated the frequency of the unknown population (Fig. 2C and G). To quantify discrepancies between the estimated and manually gated frequencies in all populations, we examined three common metrics: maximum error, Canberra distance, and intersection distance, which measure maximum deviations, the capability of capturing rare populations and accumulative errors respectively. In general, both ACDC and the phenograph clustering estimated the population frequency up to 2% maximum error of manual gating reports and 2–5% error accumulatively on these two datasets (Fig. 2D and H). However, ACDC showed a lower Canberra distance to manual gating, highlighting lower discrepancy for rare populations.

### 3.4 ACDC captures sample variations in population frequencies

**Fig. 3:**
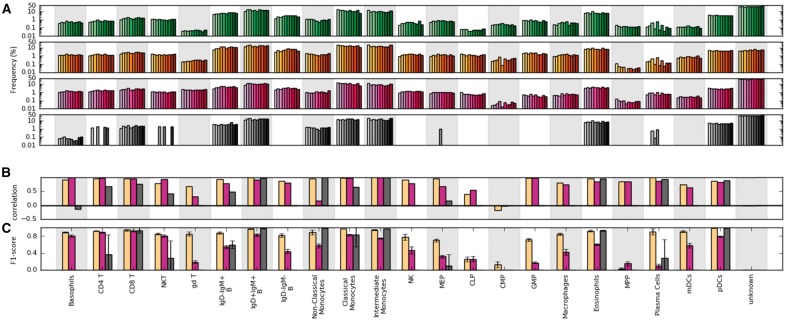
Validation on PANORAMA dataset. (**A**) Frequencies of cellular populations estimated by manual gating (green bars), ACDC (yellow bars), scored-based classification (purple bars) and phenograph clustering (gray bars). All events excluded by manual gating were labeled ‘unknown.’ (**B**) Per-cell type Pearson correlations over 10 replications. (**C**) Average F1-scores over 10 replications. Error bars represent standard deviations

In addition to evaluating the classification accuracy using data collected from one set of samples, we wondered if ACDC captured variations accurately over biological replicates in the PANORAMA dataset (Fig. 3A). We computed correlations between estimated and manually gated frequencies per cell type (Fig. 3B). ACDC achieved an average per-cell type correlation of 0.79, compared to the correlation of 0.71 for the score-based classification and 0.38 for phenograph clustering. Regarding classifying single-cell events, ACDC achieved a median F1-score of 0.88 (Fig. 3C) compared to 0.79 obtained in the original study (Samusik *et al.*, 2016), though two cell types were omitted due to the lack of defining markers when curating the input table for ACDC (see **Methods** for full details). These results confirm that ACDC more accurately captures sample variations reflected in the manually gated results.

### 3.5 ACDC discovers ambiguous populations from mass cytometry data

**Fig. 4:**
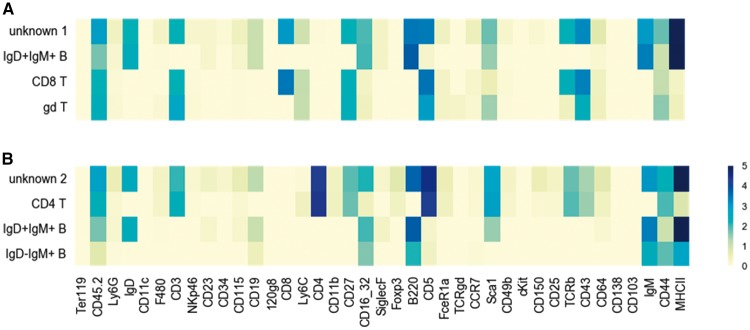
Illustration of selected unknown clusters. (**A**) Two-dimensional heatmap shows the profile of an unknown cluster sharing features of CD8+ T cells, IgD + IgM+ B cells and gamma-delta T cells (rows shown below). Colors reflect the marker intensity. (**B**) Heatmap indicates the profile of an unknown cluster sharing features of CD4+ T cells and IgD + IgM+ B cells (rows shown below). The top-3 similar canonical populations are shown right below the unknown cluster

One challenge for supervised learning approaches is the limited ability to discover categories not present in the training data. Here we demonstrate that ACDC provides insight on clusters of cells that do not fit into any of the pre-defined cell types. Specifically, 24 clusters of unknown cell types detected from the PANORAMA dataset (Supplementary Fig. S4). We found that one of the unknown clusters showed marker patterns similar to both IgD + IgM+ B-cells and CD8+ T cells (Fig. 4A). This profile suggests the unknown cluster represents some form of lymphoid cells sharing characteristics of B cells and CD8 T cells. We also found a cluster of unknown cell types that shared features of IgD + IgM+ B cells and CD4+ T cells, and cannot be easily categorized into conventional types (Fig. 4B). Though we cannot exclude the possibility these events are doublets that slipped though the pre-gating quality control carried out in (Samusik *et al.*, 2016) (Supplementary Fig. S5), these results demonstrated that ACDC can highlight ambiguous events that escaped the automated classification for further investigation. However, resolving the biological identity of these events may require utilization of collaborative evidence.

### 3.6 Robustness and computational complexity

We evaluated whether ACDC is robust to changes in the parameter tuning. ACDC uses one parameter *k* to construct nearest neighbor networks for semi-supervised classification. Table 1 shows the classification accuracy evaluated on the BMMC and AML benchmark datasets when setting *k* to 10, 20 and 30. The results are not sensitive to the parameter *k* over a 3-fold range.

**Table 1:** Computational performance of ACDC

	Accuracy (%)			Time (s)			Events
k-nn	10	20	30	10	20	30	
BMMC	92.02	92.24	92.49	245	309	376	81747
AML	98.36	98.30	98.25	884	992	1077	103184

We also examined the computational complexity of ACDC. The most expensive computational step in ACDC is the semi-supervised classification, which involves constructing and inverting a large matrix. In our current implementation, ACDC takes ∼250 and ∼900 seconds to process BMMC and AML benchmarks (**Table 1**). This computation was done on a machine with an Intel^®^ Core™ i7-6700K Processor 3.40 GHz and 16 GB RAM. By comparison, it takes ∼125 and ∼550 s to cluster the BMMC and AML datasets using Phenograph on the same machine.

## 4 Conclusion

Here we have introduced a new method called ACDC that combines profile matching and semi-supervised learning to automate the analysis and interpretation of mass cytometry data. ACDC takes advantage of biological knowledge to guide learning algorithms and creates a new framework for interpreting data from high-dimensional cytometry. By using biological knowledge as an input for the analysis, we turned the unsupervised problem of data interpretation into a semi-supervised problem of network propagation. Our results suggest ACDC reliably classifies single-cell events and aids discovery of novel cell types.

One limitation of ACDC is that each marker label is binary (present or absent). In practice, cell populations of interests are defined by intermediate markers (Guilliams *et al.*, 2014; Levine *et al.*, 2015; Ohradanova-Repic *et al.*, 2016; Rosenblum *et al.*, 2016). One possible improvement is to extend the Gaussian mixture model and consider multiple states (Chan *et al.*, 2008; Cron *et al.*, 2013), and we anticipate this development in a future study.

Given the active development of many algorithms to facilitate the processing and analysis of high-throughput cytometry data, recent efforts have also been focused on developing reproducible pipelines and frameworks (Aghaeepour *et al.*, 2013, 2016; Finak *et al.*, 2014). The introduction of a study-specific table with markers and cell labels offers a new direction toward automatic and reproducible analysis of mass cytometry data. With this easy-to-customize design, the annotation step feeds into cytometry data analysis upfront. This feature allows the cellular determinations to be reproduced or modified easily with a given cell type–marker table. Additionally, flagging ambiguous events help sift through the massive data to guide researchers for follow up on areas of quality control and process improvement, as well as the discovery of biologically relevant cell populations.

Currently, our design requires a table specified by the analyst. However, there’s no limit to what information goes into this table. Thus, it’s possible to infer a comprehensive table automatically from the complete biomedical literature mining (Courtot *et al.*, 2015; Shen-Orr *et al.*, 2009) or through a targeted query of an immunological database (Courtot *et al.*, 2015). The community has long recognized the importance of reliable immunophenotyping analysis in flow cytometry (Aghaeepour *et al.*, 2013; Finak *et al.*, 2016). Additional efforts to integrate existing tools into shared computational pipelines for better CyTOF processing and cell type enumeration are needed. With the removal of the manual processing steps that currently limit large-scale CyTOF analysis, we envision ACDC as a step toward a new paradigm of reproducible, systematic and objective immunophenotyping that fully embraces high-dimensional datasets for discovery and translation to actionable insights.

## Supplementary Material

Supplementary Data
