# Supplementary material for: Automated cell type discovery and classification through knowledge transfer
Source: Bioinformatics. 2017 Jan 31;33(11):1689–95. doi: 10.1093/bioinformatics/btx054 (PMC5447237; doi:10.1093/bioinformatics/btx054)
Supplement: Supplementary Data [file btx054_supp.docx]

Supplementary Materials

**Supplementary Table 1**. Marker profiles of cell types used for analyzing BMMC dataset.

|  | CD45 | CD45RA | CD19 | CD11b | CD4 | CD8 | CD34 | CD20 | CD33 | CD123 | CD38 | CD90 | CD3 |
| --- | --- | --- | --- | --- | --- | --- | --- | --- | --- | --- | --- | --- | --- |
| Mature CD4+ T | + | - | - |  | + | - |  |  | - |  | - |  | + |
| Mature CD8+ T | + | - | - |  | - | + |  |  | - |  | - |  | + |
| Naive CD4+ T | + | + | - |  | + | - |  |  | - |  | - |  | + |
| Naive CD8+ T | + | + | - |  | - | + |  |  | - |  | - |  | + |
| CD11bhi Monocyte | + |  | - | + | - | - | - |  | + |  |  |  | - |
| CD11b- Monocyte | + |  | - | - | - | - | - |  | + |  |  |  | - |
| Pre-B I |  |  | + |  | - | - | + | - | - |  | + |  | - |
| Pre-B II |  |  | + |  | - | - | - | - | - |  | + |  | - |
| Immature B |  |  | + |  | - | - | - | - | - |  | - |  | - |
| Mature CD38lo B |  |  | + |  | - | - | - | + | - |  | - |  | - |
| Mature CD38mid B |  |  | + |  | - | - | - | + | - |  | + |  | - |
| NK | + | + | - |  | - | - |  |  | - |  | + |  | - |
| Plasmacytoid DC | + | + | - |  | - | - | - |  | - | + | + |  | - |
| CMP |  | - | - |  | - | - | + |  | - | - | + |  | - |
| GMP |  | + | - |  | - | - | + |  | - | - | + |  | - |
| HSC |  | - | - |  | - | - | + |  | - |  | - | + | - |
| MEP |  | - | - |  | - | - | + |  | - | + | + |  | - |
| MPP |  | - | - |  | - | - | + |  | - |  | - | - | - |
| Plasma cell |  |  | + |  | - | - |  | - | - |  | + |  | - |

**Supplementary Table 2**. Marker profiles of cell types used for analyzing AML dataset.

|  | CD19 | CD4 | CD8 | CD34 | CD20 | CD45 | CD123 | CD11c | CD7 | CD16 | CD38 | CD3 | HLA-DR | CD64 |
| --- | --- | --- | --- | --- | --- | --- | --- | --- | --- | --- | --- | --- | --- | --- |
| Basophils | - |  | - | - | - |  | + | - | - | - |  | - | - | - |
| CD4 T cells | - | + | - | - | - |  | - | - |  | - |  | + | - | - |
| CD8 T cells | - | - | + | - | - |  | - | - | + | - |  | + | - | - |
| CD16- NK cells | - |  |  | - | - |  | - | - | + | - |  | - | - | - |
| CD16+ NK cells | - |  |  | - |  |  | - | - | + | + |  | - | - | - |
| CD34+CD38+CD123- HSPCs | - |  | - | + | - | - | - | - | - | - | + | - |  | - |
| CD34+CD38+CD123+ HSPCs | - |  | - | + | - | - | + | - | - | - | + | - |  | - |
| CD34+CD38lo HSCs | - |  | - | + | - | - | - | - | - | - | - | - |  | - |
| Mature B cells | + |  | - | - |  |  | - | - | - | - |  | - |  |  |
| Plasma B cells | + |  | - | - |  |  | - | - | - | - | + | - | - |  |
| Pre B cells | + |  | - | - |  |  | - | - | - | - | + | - | + | - |
| Pro B cells | + |  | - | + |  | - | - | - | - | - | + | - |  | - |
| Monocytes | - |  | - | - | - |  | - | + | - |  |  | - | + |  |
| pDCs | - |  | - | - | - |  | + | - | - |  |  | - | + | - |

**Supplementary Table 3**. Marker profiles of cell types used for analyzing PANORAMA dataset.

|  | IgD | CD11c | F480 | CD3 | CD34 | CD19 | 120g8 | CD8 | Ly6C | CD4 | CD11b | CD27 |
| --- | --- | --- | --- | --- | --- | --- | --- | --- | --- | --- | --- | --- |
| Basophils |  |  |  | - |  | - |  |  |  |  |  |  |
| CD4 T |  |  |  | + |  | - |  | - |  | + |  |  |
| CD8 T |  |  |  | + |  | - |  | + |  | - |  |  |
| NKT |  |  |  | + |  | - |  | - |  | - |  |  |
| gd T |  |  |  | + |  | - |  |  |  | - |  |  |
| IgD-IgM+ B | - |  |  | - |  | - |  |  |  |  |  |  |
| IgD+IgM+ B | + |  |  | - |  | - |  |  |  |  |  |  |
| IgD-IgM- B | - |  |  | - |  | - |  |  |  |  |  |  |
| Non-Classical MCs |  |  |  | - |  | - |  |  | - | - |  |  |
| Classical MCs |  |  |  | - |  | - |  |  | + |  | + |  |
| Intermediate MCs |  |  |  | - |  | - |  |  |  | - |  |  |
| NK |  |  |  | - |  | - |  |  | - |  |  |  |
| MEP |  |  |  | - |  | - |  |  |  |  |  |  |
| CLP |  |  |  | - | + | - | - |  | - |  |  | + |
| CMP |  |  |  | - | + | - |  |  | - |  |  |  |
| GMP |  |  |  | - | + | - |  |  | + |  |  |  |
| Macrophages |  |  | + | - |  | - |  |  | - | + |  |  |
| Eosinophils |  |  | + | - |  | - |  |  | - | - |  |  |
| MPP |  |  |  | - | + | - | - |  |  |  |  | + |
| Plasma Cells |  |  |  | - |  | - | - |  |  |  |  | - |
| mDCs |  | + |  | - |  | - |  |  | - | - |  |  |
| pDCs |  |  |  | - |  | - | + |  |  |  |  |  |

**Supplementary Table 3 (continued)**. Marker profiles of cell types used for analyzing PANORAMA dataset.

|  | CD16_32 | SiglecF | B220 | FceR1a | Sca1 | CD49b | cKit | CD150 | CD43 | IgM | MHCII |
| --- | --- | --- | --- | --- | --- | --- | --- | --- | --- | --- | --- |
| Basophils |  |  |  | + |  | + |  |  |  |  |  |
| CD4 T |  |  |  |  |  |  |  |  |  |  |  |
| CD8 T |  |  |  |  |  |  |  |  |  |  |  |
| NKT |  |  |  |  |  | + |  |  |  |  |  |
| gd T |  |  |  |  |  | - |  |  |  |  |  |
| IgD-IgM+ B |  |  |  |  |  |  |  |  |  | + |  |
| IgD+IgM+ B |  |  |  |  |  |  |  |  |  | + |  |
| IgD-IgM- B |  |  |  |  |  |  |  |  |  | - |  |
| Non-Classical MCs | + | - |  |  | - | - |  | - | + |  | - |
| Classical MCs | + |  |  |  | - | - | - | - | - |  |  |
| Intermediate MCs | + | - |  |  | - | - |  | - | + |  | - |
| NK |  |  | - | - | - | + |  | - | - |  |  |
| MEP | - | - |  |  | - | - |  | + |  |  |  |
| CLP |  |  |  |  | + | - |  |  |  |  |  |
| CMP | + |  |  |  | - | - |  | + |  |  | + |
| GMP | + |  |  |  | - | - |  | + |  |  |  |
| Macrophages | + |  |  |  | - | - |  | - |  |  |  |
| Eosinophils | + | + |  |  | - | - |  | - |  |  | - |
| MPP |  |  |  |  | + | - | + |  |  |  |  |
| Plasma Cells |  |  |  | + |  | - |  |  |  | + |  |
| mDCs | + |  |  |  | - | - |  | - |  |  | + |
| pDCs |  |  |  |  | + | - |  |  |  |  |  |


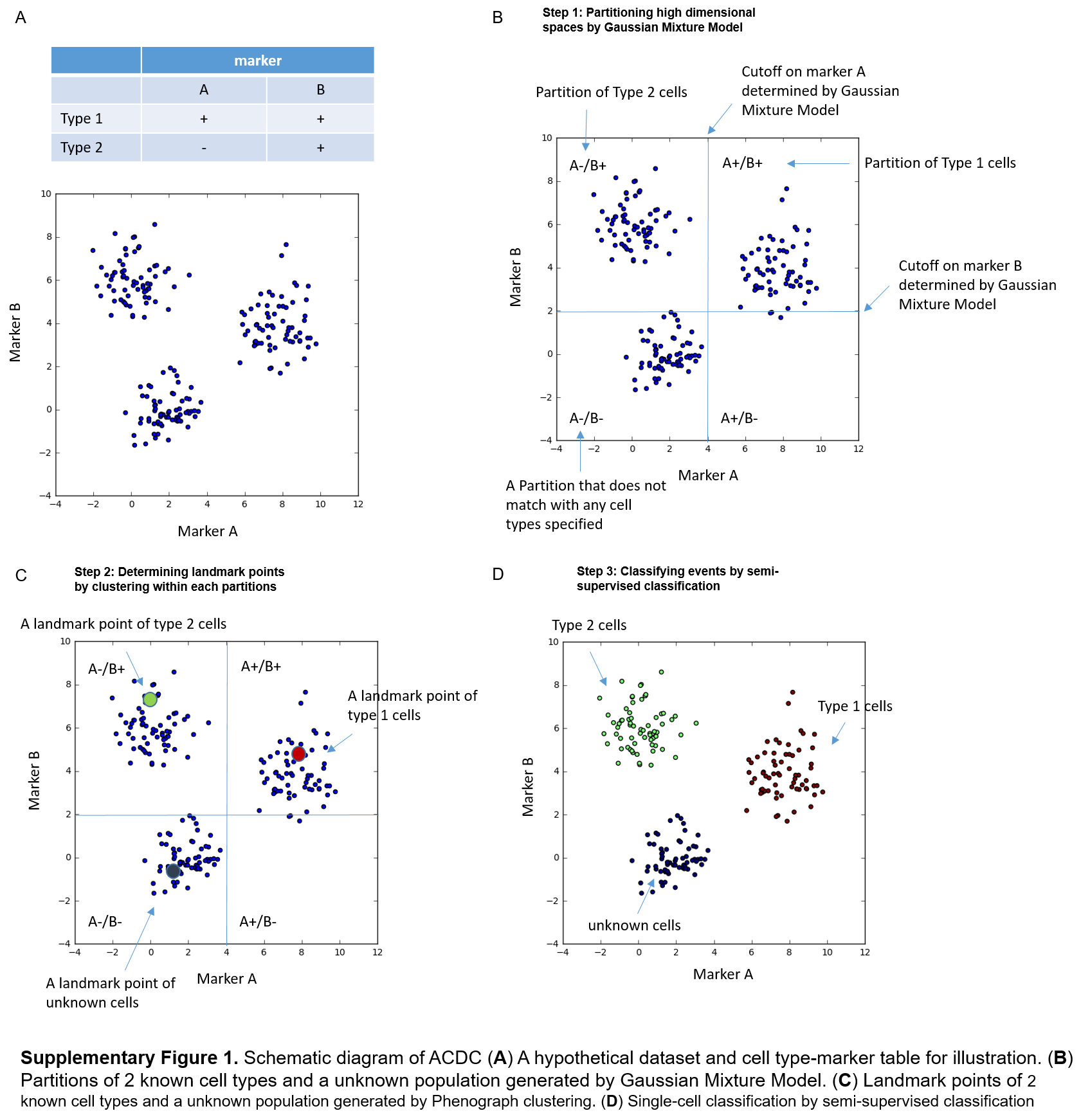


**Supplementary Figure 1.** Schematic diagram of ACDC workflow. (**A**) A hypothetical dataset and cell type-marker table for illustration. (**B**) Partitions of 2 known cell types and an unknown population generated by Gaussian Mixture Model. (**C**) Landmark points of 2 known cell types and an unknown population generated by identifying the cluster center within each partition. (**D**) Single-cell classification by semi-supervised classification.


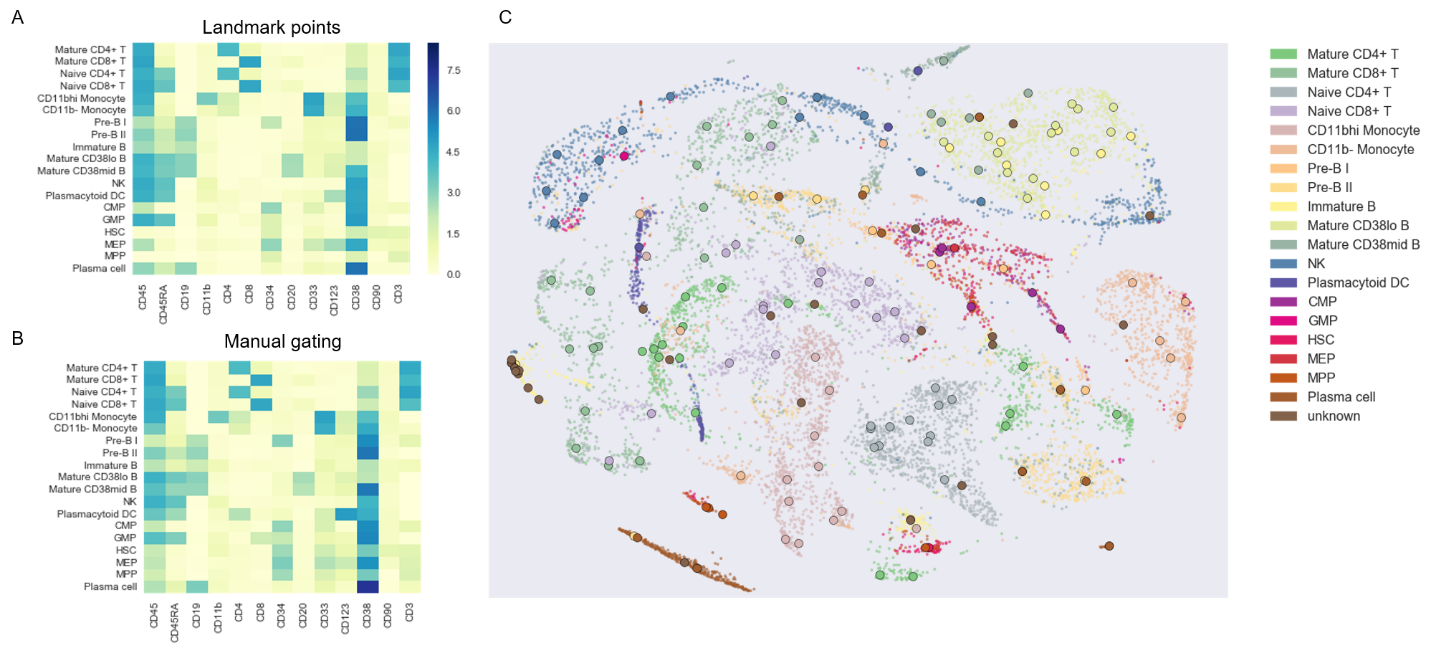


**Supplementary Figure 2**. Visualization of landmark points in BMMC dataset. Heat maps show the average marker signatures of (**A**) landmark points for each canonical cell type and (**B**) manually gated data. (**C**) Two-dimensional visualization of landmark points (large circles) along with up to 1000 randomly sampled events from each manually gated cellular population. Each dot represents a single cell. The tSNE algorithm was used to reduce the dimensionality of the data.


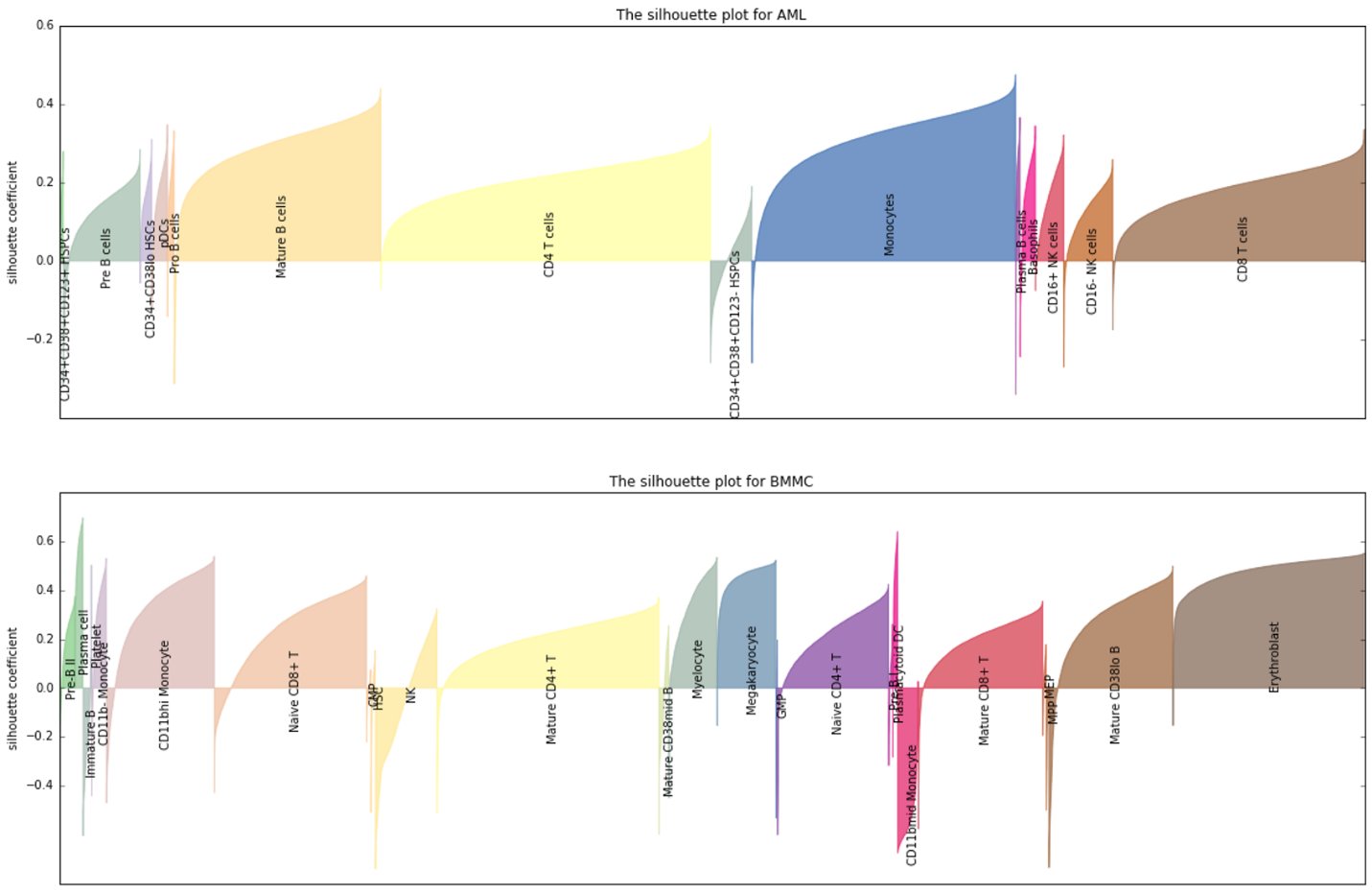


**Supplementary Fig. 3**. Silhouette plot of manually gated populations. Each colored region represents the sorted Silhouette coefficients for all samples in the labeled population. High values of the silhouette coefficient indicate an event is surrounded by events of the same type.

**
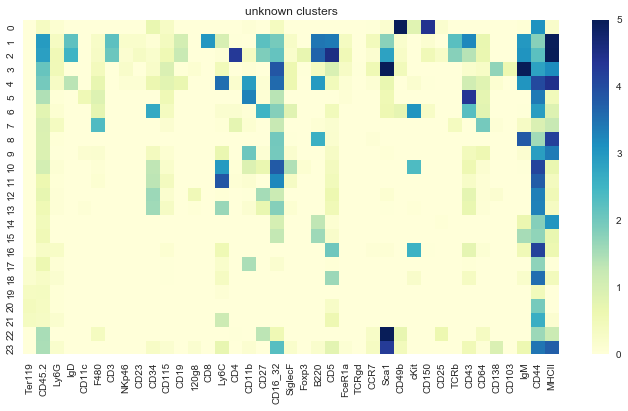
**

**Supplementary Fig. 4**. Marker signatures of 24 unknown clusters detected from PANORAMA dataset.


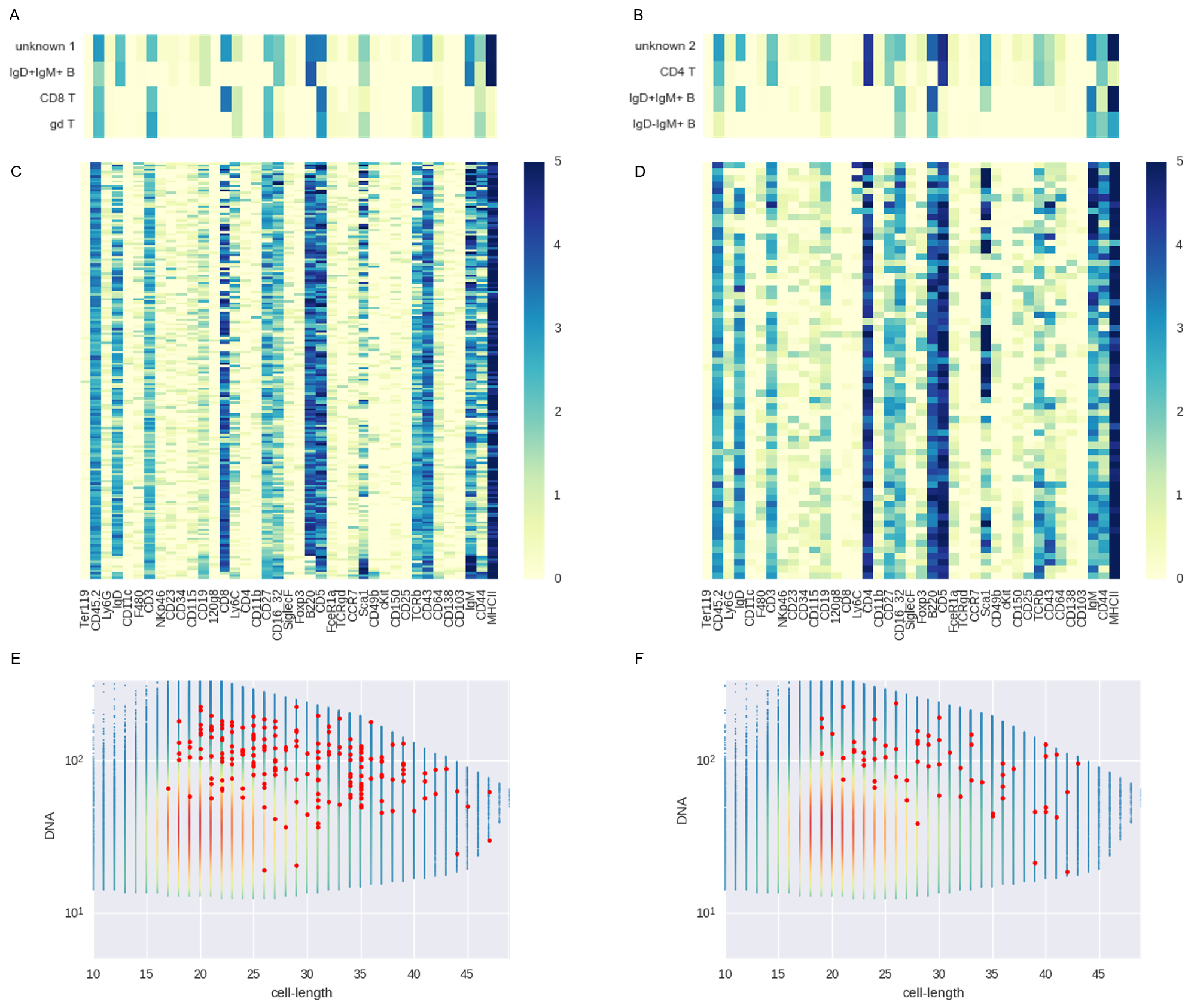


**Supplementary Figure 5.** Illustration of selected unknown clusters. (**A**) The average profile of an unknown cluster sharing features of CD8 T cells and IgD+IgM+B cells. (**B**) The average profile of an unknown cluster sharing features of CD4 T cells and IgD+IgM+B cells. (**C & D**) Profiles of the raw events associated with each cluster in (**A**) and (**B**) respectively. (**E & F**) Scatter plot of cell-length versus DNA. All events pre-processed to remove doublets, dead cells, and select leukocytes (Samusik *et al.*, 2016). Red circles represent raw events listed in (**C**) and (**D**), respectively. The background scatterplot represents the distribution of all events. The Colors indicates the total number of cells within a bin.

**References**

Samusik, N. *et al.* (2016) Automated mapping of phenotype space with single-cell data. *Nat Meth*, **13**, 493–496.
